# Effects of Iodine Doping on Optoelectronic and Chemical Properties of Polyterpenol Thin Films

**DOI:** 10.3390/nano7010011

**Published:** 2017-01-13

**Authors:** Kateryna Bazaka, Mohan V. Jacob

**Affiliations:** 1 School of Chemistry, Physics and Mechanical Engineering, Queensland University of Technology, Brisbane, QLD 4000, Australia; 2 CSIRO-QUT Joint Sustainable Materials and Devices Laboratory, Commonwealth Scientific and Industrial Research Organisation, P.O. Box 218, Lindfield, NSW 2070, Australia; 3 College of Science and Engineering, James Cook University, Townsville, QLD 4814, Australia; mohan.jacob@jcu.edu.au

**Keywords:** plasma polymerization, iodine doping, thin film, optical properties, organic polymer

## Abstract

Owing to their amorphous, highly cross-liked nature, most plasma polymers display dielectric properties. This study investigates iodine doping as the means to tune optoelectronic properties of plasma polymer derived from a low-cost, renewable resource, i.e., *Melaleuca alternifolia* oil. In situ exposure of polyterpenol to vapors of electron-accepting dopant reduced the optical band gap to 1.5 eV and increased the conductivity from 5.05 × 10^−8^ S/cm to 1.20 × 10^−6^ S/cm. The increased conductivity may, in part, be attributed to the formation of charge-transfer complexes between the polymer chain and halogen, which act as a cation and anion, respectively. Higher levels of doping notably increased the refractive index, from 1.54 to 1.70 (at 500 nm), and significantly reduced the transparency of films.

## 1. Introduction

The employment of plasma polymerization techniques for synthesis of high-performance organic thin film materials has been investigated by many authors because these techniques allow the polymerization of a range of organic compounds, including those that may not necessarily polymerize through conventional thermo-chemical pathways [1,2,3]. Polymers, fabricated using RF plasma polymerization, are smooth, pinhole-free, and uniform, with chemical and physical properties that can be tailored by controlling such deposition parameters as pressure, RF power, monomer flow rate, and time of fabrication [4,5,6,7,8]. Owing to their high-resistivity and high dielectric breakdown strength, organic polymer materials have found numerous applications, such as thin film insulators and capacitors for integrated microelectronics, passivation layers in integrated circuits, and protective coatings. Polymer materials manufactured from renewable natural sources, such as essential oils, offer an additional advantage of being environmentally sustainable compared to polymer materials derived from fossil fuels [9,10]. Much research has been devoted to enhancing the electrical conductivity of organic polymers, primarily through doping them with appropriate electron donors (such as alkali metals) or acceptors (such as iodine or arsenic pentafluoride) [11]. Iodine doping has been studied principally because of the significant effect an introduction of this doping agent has on the electrical and dielectric properties of polymer thin films, including an increase in electrical conductivity due to oxidation of the polymer and a change in polymer charge-storing capacity [12].

In this paper we report on the effect that in situ iodine doping has on optoelectronic and chemical properties of the plasma-polymerized terpinen-4-ol thin films, herein called polyterpenol. Polyterpenol is fabricated from non-synthetic mono terpene alcohol, a major constituent of *Melaleuca alternifolia* oil, by way of radio frequency polymerization. With a refractive index slightly above that of glass, transparency to the visible region, very smooth surface, and high chemical inertness and thermal stability, polyterpenol films have prospective applications in organic optoelectronics as encapsulating layers for the organic circuitry. Polyterpenol has also been demonstrated as an appropriate insulating material to improve the output characteristics of organic field effect transistors (OFET) and is a prospective candidate for full flexible organic electronic circuits [5,13,14].

## 2. Results and Discussion

### 2.1. Chemical Properties

**Figure 1:**
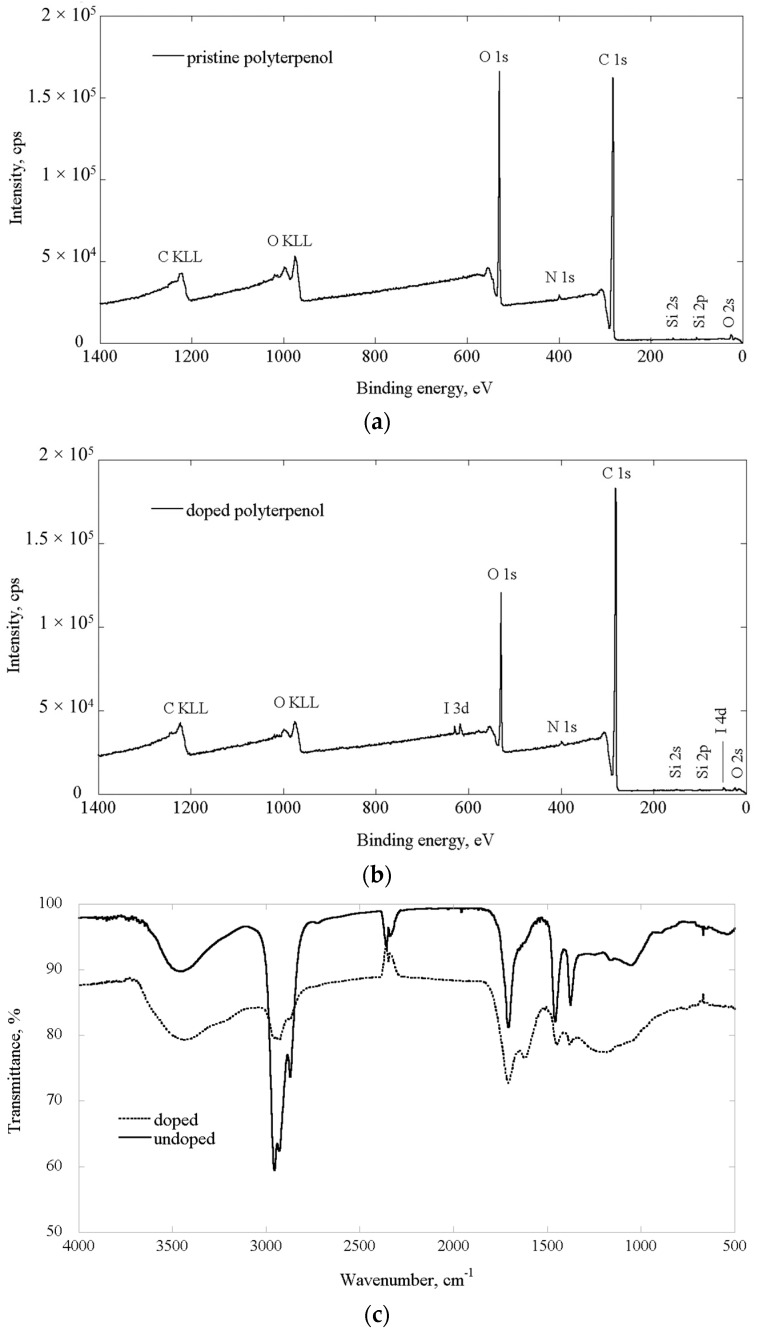
Chemical properties of pristine and iodine-doped polyterpenol samples: XPS spectra for pristine (**a**) and doped (**b**) samples; and (**c**) FTIR spectra.

Fourier transform infrared spectroscopy (FTIR) and X-ray photoelectron spectroscopy (XPS) were used to analyze the changes in chemical structure of polyterpenol films as a consequence of doping. XPS survey spectra collected for pristine and iodine-doped polyterpenol samples deposited under the same conditions are depicted in Figure 1, and the atomic fractions of elements present in the films are summarized in Table 1. Similar to pristine polyterpenol film, the iodine doped polymers are hydrocarbon in nature, with low iodine concentration. Si and N atoms detected by the XPS are likely the contaminations that have originated from glass and air, respectively. This is in agreement with our previous findings, where trace amounts of Si and Zn were both attributed to the contribution from the substrate [15]. In the doped film, there is a significant reduction in the oxygen to carbon ratio compared to pristine polyterpenol. Such a change is likely to be associated with the introduction of an impurity into the polymer structure via the elimination of oxygen, possibly through the formation of highly oxidizing iodine compounds.

In the FTIR spectra of the monomer and pristine polymer sample (Figure 1) the previously reported peaks were observed [4]. The FTIR absorption bands and the suggested groups for pure and iodine-doped polyterpenol are presented in Table 1. Introduction of iodine resulted in the significant reductions in the intensity of bands associated with methyl and methylene functional groups. The relationship between intensities of methyl (1380 cm^−1^) and methylene/methyl (1450 cm^−1^) bands indicates chain branching and suggests that, similar to pristine polyterpenol, iodine-doped film is comprised mostly of short polymer chains rather than long linear backbone structures [16]. FTIR spectra for a number of iodine-doped samples contained peaks within 600–480 cm^−1^ region, which correspond to aliphatic C–I stretch, and may suggest incorporation of iodine atoms into the backbone of the polymer, similar to that observed in co-polymers of pyrrole and ethylenglycol doped with iodine during plasma polymerization [17]. All FTIR spectra contained peaks within 2500–2000 cm^−1^ region. These peaks are not inherent to the polymer, and arise as a result of inadequate correction for the background CO_2_ and H_2_O present in the ambient air. Similar artefacts have been observed previously [4], and were absent when a different FTIR instrument was used, whereas the position and magnitude of other peaks inherent to the polymer were preserved.

The shifts observed in C–H frequencies and skeletal C–C vibration bands can be attributed to high electronegativity of iodine atoms that can have marked effect on the spectrum of neighboring group frequencies. Integration of iodine into the polyterpenol molecular structure, most likely by abstraction of H, is further supported by the presence of the group frequency associated with conjugated C=C bond. Considering the complex chemical structure of polyterpenol thin film and presence of a large number of residual radicals commonly observed in plasma polymers, several different reactions with iodine are likely to occur. Formation of iodine oxidants during polymerization can lead to oxidation of alcohol moieties to ketones. Furthermore, light-induced homolytic dissociation of iodine may lead to formation of additional iodine radicals that can, in turn, initiate hydrogen abstraction. Therefore, in situ iodine doping results in a change in structure of plasma polymerized terpinen-4-ol thin film samples.

### 2.2. Optoelectrical Properties

**Figure 2:**
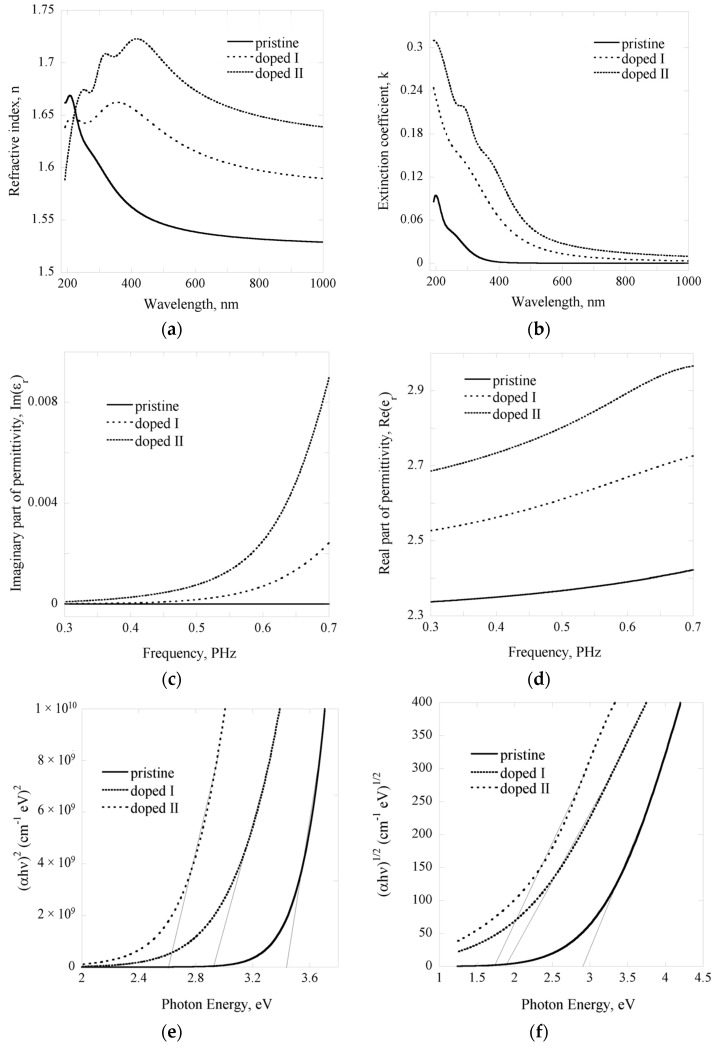
Optical properties of pristine and iodine-doped polyterpenol samples obtained using ellipsometry: (**a**) refractive index; (**b**) extinction coefficient; (**c**) real and (**d**) imaginary parts of permittivity as a function of frequency; (**e**) direct and (**f**) indirect allowed transition energies of pristine and iodine-doped polyterpenol films.

Spectroscopic ellipsometry and UV-VIS studies were carried out on the polyterpenol thin films deposited on glass substrates to elucidate the effect of iodine doping on the thickness and optical properties of the thin polymer films. Spectroscopic ellipsometry measures functions of the Fresnel reflection coefficients (*Ψ*, Δ) which are then parameterized as a function of wavelength in order to obtain thin film parameters, such as film thickness, surface roughness, refractive index, and extinction coefficient spectral dependencies, dielectric functions, and others [18,19]. The relationship between *Rp* and *Rs* Fresnel coefficients (for the *p*- and *s*-polarizations respectively) and the complex reflection coefficient (ρ) can be expressed through:

(1) $$\rho =\frac{R_{p}}{R_{s}}=\tan\left(\Psi \right)e^{i\Delta }=\frac{\left|r_{p}\right|}{\left|r_{s}\right|}e^{i\Delta }$$

where *r_p_* and *r_s_* are complex Fresnel coefficients, Δ a phase shift between *s* and *p* polarized waves [20]. Gaussian (harmonic) oscillators are suggested for fitting conjugated polymer data in order to obtain the values for the film optical constants [19,21]. Mean-square error values of below 2 were achieved in the modelling process. Comparisons between the refractive index, extinction coefficient, and transmission profiles of the pristine and iodine-doped polyterpenol thin films are presented in Figure 2. Higher levels of doping notably increased the refractive index of the thin films, from 1.54 to 1.70 at 500 nm wavelength, and significantly reduced the transparency of films, owing to the strong absorption of visible light. 

For the dielectric function, the real and imaginary terms can be expressed as follows:

(2) $$\varepsilon_{2}\left(E\right)=Ae^{-{\left(\frac{E-E_{n}}{\sigma }\right)}^{2}}-Ae^{-\left(\frac{E\;+\;E_{n}}{\sigma }\right)}$$

(3) $$\varepsilon_{1}\left(E\right)=\frac{2}{\pi }P\int_{E_{g}}^{\infty }\frac{\xi \varepsilon_{2}\left(\xi \right)}{\xi^{2}-E^{2}}d\xi$$

where *A* is the amplitude, σ the full width at half maximum, and *E_n_* the peak position [22]. From ellipsometric studies, in the wavelength region of 200–1000 nm, the real part of permittivity (*k*) was found to be between 2.34 and 2.78 for pristine and between 2.52 and 2.97 for iodine-doped polyterpenol samples. This is similar to the values obtained for plasma polymerized linalyl acetate thin films [23]. At frequencies above 10 Hz, electronic phenomena contribute to approximately 70%–80% of the determined dielectric constant, with ionic (*ε*_ionic_) and orientational (*ε*_orientational_) phenomena in the bulk of the film accounting for the remainder [23]. Owing to high level of cross-linking in plasma polymer films, the molecular mobility in these films is restricted, which, in conjunction with low ionic polarisation of molecular structures in the polymer, results in relatively low values of *ε*_ionic_ and *ε*_orientational_ [24]. Indeed, plasma polymers fabricated under low power or pulsed power conditions, those that are generally associated with lower degree of cross-linking and thus higher mobility of polymer chains, tend to display higher dielectric constant [23,24,25]. For pristine polyterpenol thin films, the results were in accord with previously reported complex permittivity studies that found polyterpenol as a potential low-k material [26]. 

The optical band gap of the polymer thin films were determined from absorption data obtained using UV-VIS spectroscopy and ellipsometry, and also by fitting a Tauc-Lorenz oscillator model to the ellipsometric data collected for the film samples. In amorphous semiconductors, the photon absorption follows the Tauc relation [27]:

(4) $$\alpha h\nu =B{\left(h\nu -E_{g}\right)}^{n}$$

where *α* is the absorption coefficient, *hν* the photon energy, *B* a constant dependent on the length of localized state tails, and *n* is determined by the nature of the electronic structure of the material, and *E_g_* the optical band gap [28]. Parameter *n* can take values of ½ for direct, and 2 for indirect, transition; the *n* value of 2 is frequently chosen for amorphous materials and indicates a parabolic function for the density of states distribution in the band tails [29,30]. Due to high degree of disorder pertinent to the structure of plasma polymers, the value of the optical gap will diverge from the energy gap value by the width of the range of localized states in the valence and conduction band [31,32].

Absorption coefficient data which resulted from the modelling of optical constants (*n*, *k*) was used to estimate the optical band gap values for pristine and iodine doped polyterpenol. As shown in Figure 2, transition energies were estimated by plotting (*αhν*)^2^ and (*αhν*)^1/2^ as a function of photon energy (*hν*) and extrapolating the linear section of the curve to the abscissa, with the intercept denoting the values for the direct and indirect transition energy gaps. UV-VIS absorbance data was treated in a similar fashion to confirm the results (data not shown here). The results showed consistency between estimation methods and are outlined in Table 2. Introduction of iodine reduced the indirect transition band gap of polyterpenol from 2.82 to 1.64 eV, the reduction attributed to the incorporation of iodine into polymer chain, as confirmed by FTIR and XPS studies, and in so doing extending the density of states more into the visible region of the electromagnetic spectrum. In addition, the increased absorption can be attributed to an increase in C=C bonds in the iodine-doped polyterpenol film layers compared to pristine polyterpenol.

Polyterpenol has a low electrical conductivity due to the weak van der Waals forces, with the variable-range hopping as the dominant charge transport mechanism due to strong localization of the charge carriers typical for the amorphous organic polymer [33]. Changes in electrical properties of doped amorphous materials, such as plasma polymers, are primarily related to the change in the gap-state occupation, rather than an increase in the band state electron or hole density characteristic of doped crystalline materials. In its doped state, the electrical activity of iodine atoms stems from a complex reaction with both dangling bonds D° and hydrogen atoms of polyterpenol chain, and the generation of an additional dangling bond state D^+^ [34]. Hence, as well as free charge carriers, iodine doping generates relatively deep localized states in the polyterpenol, in addition to the intrinsic density of states distribution [35]. Such changes in the gap-state density cause the shift in the Fermi level and, hence, the electrical conductivity of polyterpenol thin films.

**Figure 3:**
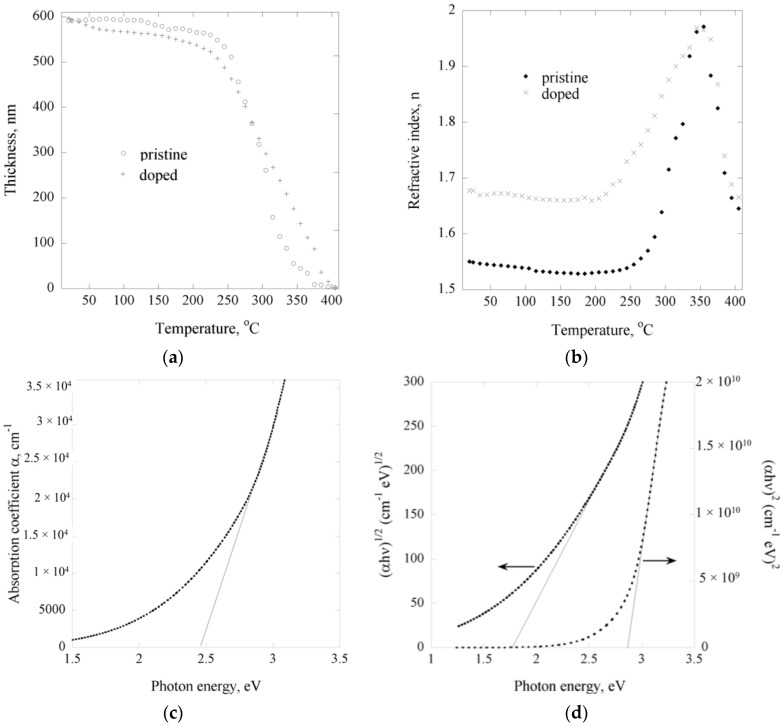
(**a**) Film thickness and (**b**) dynamic refractive index of pristine and iodine-doped polyterpenol film as a function of annealing temperature; (**c**) band gap and (**d**) direct allowed and indirect allowed transition energies of iodine doped polyterpenol thin films heat treated at 100 °C for 5 h.

Spectroscopic ellipsometry was used to investigate the stability of the iodine-doped polyterpenol thin films. Changes in thickness and optical properties of the samples were monitored as the films were annealed to 405 °C, at 10 °C steps. To reach thermal equilibrium between the stage and the samples, each measurement was taken at the end of 5 min intervals. Previous studies have shown that pristine polyterpenol films fabricated under similar RF power conditions possess two distinct regimes of thermal decomposition, with the first regime commencing at approximately 100 °C and characterized by minor thickness reduction attributed to desorption of low molecular weight species and recombination of free trapped radicals. Iodine doped samples revealed a slightly more pronounced weight loss over the same temperature range, as demonstrated in Figure 3. 

Doped polyterpenol were subjected to 5 h of annealing at 100 °C and their absorption spectrum recorded, as presented in Figure 3c. It was found that the indirect allowed transition band gap increased slightly, from 1.64 eV pre-treatment to 1.74 eV post-annealing, the increase was attributed to desorption of loosely bonded iodine atoms from the surface of the film, similar to in situ iodine-doped plasma polymerized polyaniline [36]. Subsequent heat treatment at 100 °C of these samples did not result in any further change in the band gap, an indication that the change in the optical properties of iodine doped polyterpenol was stable and permanent, and is the result of iodine atoms being incorporated into the structure of the polymer.

Unlike pristine polyterpenol films that remain relatively stable in terms of their optical properties up to 250 °C, iodine-doped samples commence a second regime of decomposition at around 200 °C with a significant increase in refractive index, as shown in Figure 3b. As discussed earlier, integration of iodine into the polyterpenol structure was likely to involve H abstraction and formation of highly reactive iodine oxidants. Increased temperature combined with light-induced homolytic dissociation of iodine would enhance the mobility and reactivity of such species, with new reaction pathways being initiated. 

In addition, pristine polyterpenol films contain a significantly larger proportion of C–H bonds which have higher dissociation energy compared to oxygen containing moieties and, hence, would contribute to thermal stability of the films. Over the temperature range of 200 °C to 405 °C, doped films undergo a phase change which is reflected in a rapid increase in refractive index and significant reduction in film thickness. Similar to polyvinyl alcohol and polyvinyl acetate, polyterpenol samples are believed to experience non-radical depolymerization via hydrogen abstraction [37], and scission of weaker C–H bonds via radical transfer to the tertiary carbon atom followed by the scission of a C–C bond in the β-position which is a common thermal degradation pathway in cross-linked and branched plasma polymers.

### 2.3. Electrical Studies

**Figure 4:**
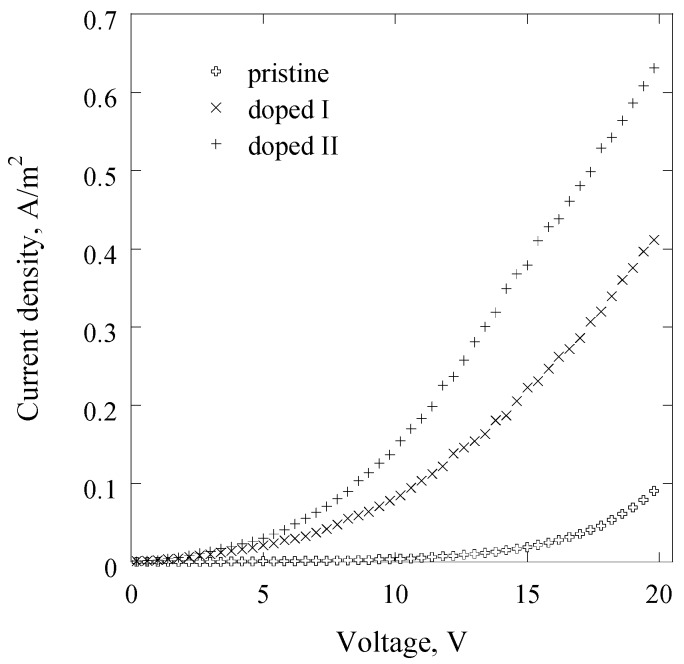
Current density, *J*, of devices containing pristine and iodine-doped polyterpenol with an applied voltage between 0 V and 20 V.

Current density–voltage relationships of the pristine and doped thin films of approximately 350 nm in thickness sandwiched between Al as electrodes are shown in Figure 4. While the *J*–*V* curves of doped samples are approximately the same shape, having two sections of different gradients in the low and high voltage regions, the *J*–*V* curve for the device containing pristine polyterpenol is distinct. 

Ability to determine a predominant mechanism of conduction in insulating polymer thin films relies on the dependence of the current density on such parameters as applied voltage, film thickness, temperature, and so on. For instance, *n* ≥ 2 generally indicates a possibility of a space charge limited conduction mechanism [38]. A linear dependence of ln*J*–ln*V* also suggests this mechanism, whereas a linear dependence of ln*J*–*V*^0.5^ indicates a possibility of Schottky or Poole–Frenkel conduction [39]. Furthermore, for space charge limited conduction mechanism, a thickness dependence of conductivity should be satisfied in the form *J* ∝ *d*^−*l*^, where *l* is a parameter associated with the trap distribution and should be *l* ≥ 3 in the presence of traps [23]. On the other hand, if Poole-Frenkel conduction or Schottky emission is assumed, the current density should obey log*J* ~ *d*^−1/2^ at a fixed voltage. The general expression for both Schottky and Poole–Frenkel type conductions is [40]:

(5) $$J=J_{0}\exp\left(\frac{\beta F^{1/2}-\phi }{kT}\right)$$

where *J*_0_ is the low field current density, *F* the applied electric field, *k* the Boltzmann constant, *T* the absolute temperature, *β* the coefficient of the static electric field, and *φ* the ionization energy of localized centers and Coulomb barrier height of the electrode polymer interface in the case of Poole–Frenkel and Schottky mechanisms, respectively. The field lowering coefficient *β* are known as *β*_S_ and *β*_PF_ in the case of Schottky and Poole–Frenkel conductions, respectively, and can be expressed as [41]:

(6) $$\beta_{\mathrm{PF}}=2\beta_{S}=2{\left(\frac{e^{3}}{4\pi \varepsilon_{0}\varepsilon_{r}}\right)}^{1/2}$$

where *e* is the electronic charge, *ε*_0_ the permittivity of free space, *ε_r_* the dielectric constant of the bulk polymer material at high frequencies. By comparing theoretical *β*_S_ and *β*_PF_ with an experimentally-attained *β* coefficient, Schottky and Poole–Frenkel mechanisms can be differentiated [25]. The following relation was used to determine *β*_exp_:

(7) $$\beta_{\exp}=skTd^{1/2}$$

where *s* is the slope of the linear section of ln*J*–*V*^1/2^ characteristic:

(8) $$s=\frac{\Delta \ln J}{\Delta \ln V^{1/2}}$$

**Figure 5:**
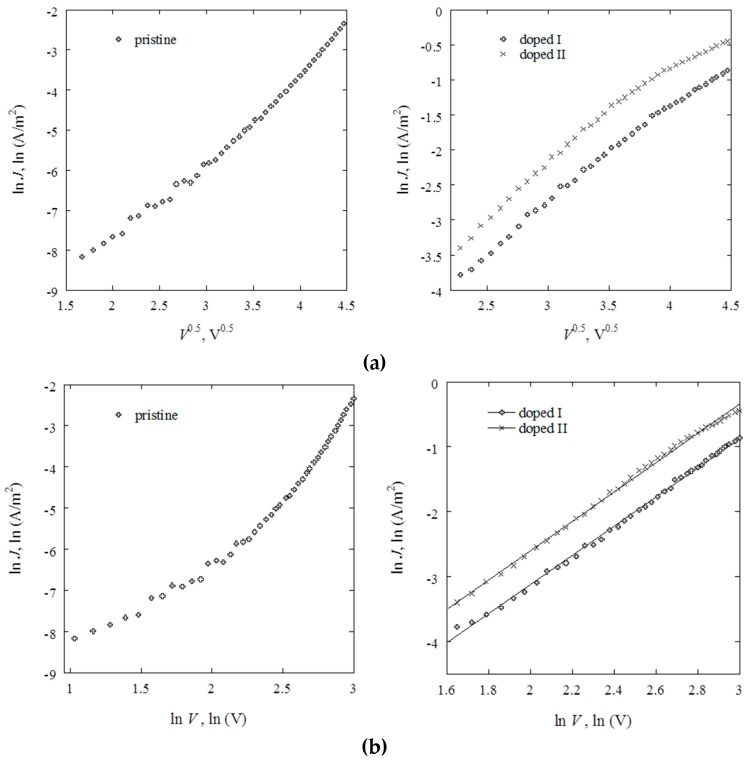
(**a**) Schottky/Poole–Frenkel and (**b**) space charge limited conduction mechanism fits to *J–V* curves in the high-field region for pristine and iodine-doped polyterpenol.

In the low field region of 0–2.5 V, the *J–V* characteristic of pristine polyterpenol can be described by a power law relation, where *J* ∝ *V^n^*, and *n* = 0.94. Such value of *n* indicates an approximately ohmic conduction mechanism [42]. Exceeding 2.5 V, the value of *n* is 2.05 and 4.68 for 2.5–10 V and 10–20 V applied voltage, respectively, indicating non-ohmic charge transport for these regions. Although *n* > 2 suggests a possibility of space charge limited conduction, fitting the *J*–*V* data, the relationship is more linear in ln*J*–*V*^0.5^ plot, suggesting Schottky or Poole-Frenkel conduction (Figure 5). This agrees well with previous studies that demonstrated an approximately Ohmic conduction in the lower applied field region, and Schottky emission in 2.5–6 V region for pristine polyterpenol. Schottky conduction was also observed in plasma polymers fabricated from lemongrass essential oil [43], cis-β-ocimene [42], γ-terpinene [25], and linalyl acetate [23].

Deposited in the glow region, plasma polymers may contain a significant concentration of trapping sites [23]. However, the value of parameter l of less than 3 required in space charge limited conduction eliminates this possibility. Then, the experimental values for *β*_exp_ were determined as 1.44 × 10^−5^ and 4.32 × 10^−5^ eV·m^1/2^·V^−1/2^ for 2.5–10 V and 10–20 V, respectively. Using the dielectric value *ε_r_* = 2.8 obtained at high frequencies using spectroscopic ellipsometry, *β*_S_ and *β*_PF_ were calculated as 2.27 × 10^−5^ and 4.54 × 10^−5^ eV·m^1/2^·V^−1/2^, respectively. It is likely that in 2.5–10 V region, the dominant transport is Schottky emission, whereas in 10–20 V applied field region, Poole–Frenkel mechanism may dominate.

Similar to pristine polyterpenol, the *J*–*V* curves of lightly doped samples were also described by the power law relation *J* ∝ *V^n^* in the lower power region, 0–5 V, with *n* of 1.08, suggesting an ohmic conduction mechanism. In the same region, strongly doped samples were described by *J* ∝ *V^n^*, with *n* = 1.33, indicating Schottky/Poole–Frenkel mechanisms as likely. In the higher applied voltage region, doped samples had *n* of 2.25 and 2.27 for lower and higher doping levels, indicating a possibility of space charge limited mechanism. A plot of ln*J*–ln*V* in this region is more linear compared to ln*J*–*V*^0.5^, advocating in favour of space charge limited conduction.

The conductivity of the samples was determined as a reciprocal of resistivity *ρ* calculated as:

(9) $$\rho =\frac{\left(\frac{V}{I}\right)A}{d}$$

where ρ is the resistivity of the material, *A is* the device surface area, *d* is the thin film thickness, *V* is the measured voltage, and *I* is the measure current. The conductivity of undoped polyterpenol was estimated to be ~ 5.05 × 10^−10^ Ω^−1^·m^−1^, higher than that of plasma polymers fabricated from another monoterpene γ-terpinene (1.02 × 10^−13^–1.39 × 10^−12^ Ω^−1^·cm^−1^) [25]. In general, plasma polymers from essential oil-derived terpenes, including cis-β-ocimene and linalyl acetate, have conductivities in the range of 10^−12^–10^−11^ Ω^−1^·m^−1^ [23,42]. This is lower in comparison to other amorphous, plasma-deposited insulators, such as those from thiophenes and polyanilines, which have the conductivity on the order of 10^−4^–10^−8^ Ω^−1^·m^−1^ and 10^−6^–10^−8^ Ω^−1^·m^−1^, respectively [44,45]. In plasma polymerized thiophene films, conductivity was found to decrease significantly with increasing deposition power density, which was attributed to an increase in monomer fragmentation, and corresponding loss of the conjugated structure of the thiophene monomer, and the chemistry of reaction products [44].

Doping increases the conductivity polyterpenol to 7.74 × 10^−9^ Ω^−1^·m^−1^ and 1.20 × 10^−8^ Ω^−1^·m^−1^ for increasing doping levels (calculated at 20 V). For comparison, plasma polymers deposited from iodothiophene have been reported to have conductivity of 1.1 × 10^−6^ Ω^−1^·m^−1^ [44]. Similarly, plasma-polymerized thiophene doped with iodine vapours had conductivities on the order of 10^−5^–10^−6^ Ω^−1^·m^−1^ [46]. When polyaniline plasma polymers were synthesized with I_2_, the electric conductivity ranged from 10^−4^ to 10^−11^ Ω^−1^·cm^−1^ [45].

### 2.4. Surface Properties

Atomic force microscopy and nanoindentation analyses were executed to highlight the changes in physical and surface properties of the polymer films while contact angle measurements are conducted to evaluate wettability of the polymer samples. 

**Figure 6:**
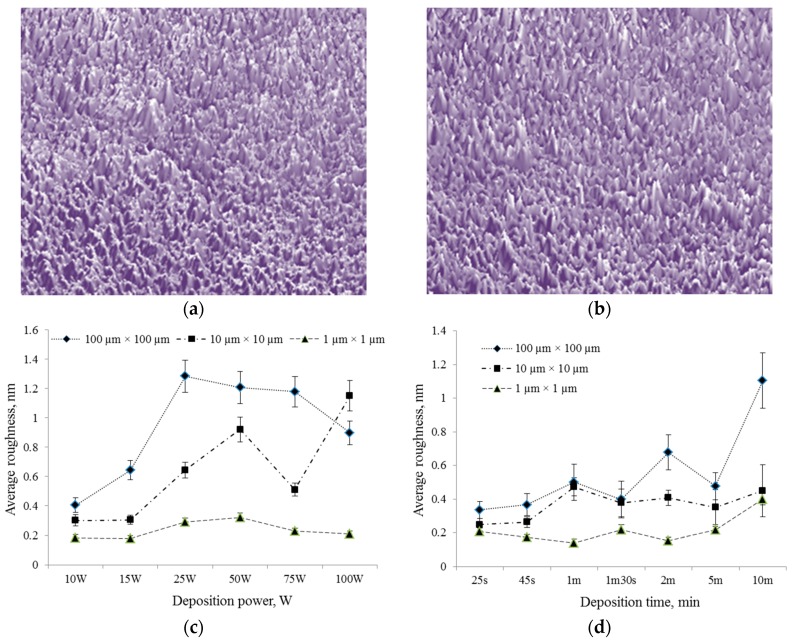
Representative atomic force microscope (AFM) images of (**a**) pristine and (**b**) iodine-doped polyterpenol thin films deposited on glass substrates. Evolution of average surface roughness of iodine-doped films as a functions of (**c**) deposition power when deposition time is fixed at 10 min, and (**d**) as a function of deposition time when deposition power is fixed at 25 W.

Examination of the surface topography of the pristine and iodine doped polyterpenol thin films showed that the surfaces were smooth, defect free and uniform (Figure 6, Table 3). The maximum peak height (*R_max_*), surface skewness (*R_skw_*), and coefficient of kurtosis (*R_kur_*) increased from 5.4 nm, 0.22, and 1.65 for pristine to 6.8 nm, 0.76, 2.51 for iodine-doped polyterpenol, respectively. Such values of *R_skw_* and *R_kur_* indicate a disproportionate number of peaks and a well spread out height distribution for the surface. The average roughness (*R_a_*) and RMS (*R_q_*) parameters of iodine doped films were found to be similar to the values for pristine polyterpenol, 0.56 nm and 0.42 nm for *R_a_* and *R_q_* respectively. Increasing the applied power or deposition time did not significantly increase the average surface roughness of iodine-doped films when examined at nanoscale (1 µm × 1 µm).

Surface wettability studies were performed using water, diiodomethane and ethylene glycol. The average contact angle reduced from 68.7° to 61.6° for water, 71.63° to 47.1° for glycerol, and from 38.1° to 28.4° for diiodomethane, for pristine and iodine doped films, respectively. Increased hydrophilicity of doped samples was attributed to a significantly larger number of polar functional groups exposed to the top of the polymer surface. Contact angle measurements were also conducted on a number of iodine-doped samples stored for 60 days in an ambient environment, with an approximately 10% increase in respective contact angles for water and glycerol compared to those pertaining to freshly-deposited doped surfaces. Plasma polymerized thin films are frequently characterized by a significant number of radicals trapped within the bulk of the polymer. After the deposition, these radicals continue to react with one another, the polymer molecules, and the ambient environment. Rapid oxidation of polymer upon exposure to the air is common for plasma polymers, and results in the formation of oxygen-containing groups on the surface of the films, rendering the film more hydrophilic. Overtime, however, the polar moieties have a propensity to migrate into, or re-orientate themselves toward, the bulk of the film in an attempt to minimize the interfacial tension at the polymer/air interface. Such change in surface configuration can account for the change in the contact angle behavior of doped polymer films with time. Under similar exposure conditions, the surface configuration of pristine polyterpenol remains relatively constant, due to initially lower surface tension and consequent lower interfacial tension between the surface of polyterpenol and ambient air.

The average contact angle for each liquid was used to calculate the surface free energy and its components, based on the Lewis acid/base method [47], and the results are summarized in Table 3. As is the case with pristine polyterpenol films, iodine doped samples were found to be monopolar in nature, largely manifesting electron-donor properties [48]. However, the electron-acceptor fraction of the surface tension for iodine doped samples was significantly higher than that of pristine polyterpenol, due to the presence of iodine atoms. As a monopolar solid, the iodine polyterpenol is likely to interact with polar solvents, such as water. Over time, the polar components of surface tension and the total surface tension of the film surface decrease, hence, lowering the solvophilic response of the surface to polar solvents.

## 3. Materials and Methods

**Figure 7:**
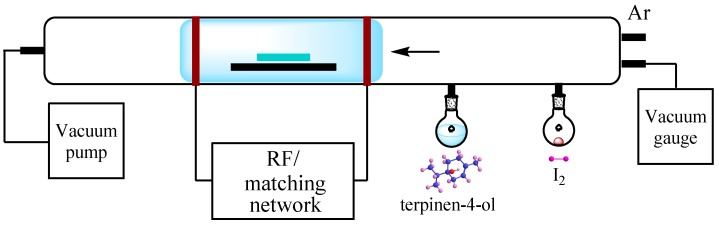
Schematic of the plasma polymerization system. Inter-electrode distance is 10 cm. Deposition takes place within the plasma glow, with terpinen-4-ol and iodine vapours released into the glass chamber concurrently, in the absence of heating or carrier gas.

Pristine and iodine doped polyterpenol thin films were fabricated from non-synthetic terpinen-4-ol monomer (distilled by Australian Botanical Products, Hallam, Australia) using the experimental setup described in [49]. RF energy (13.56 MHz) was delivered into the deposition chamber by means of capacitively-coupled copper electrodes (Figure 7). Substrates (superwhite glass slides and KBr pallets) were cleaned according to the procedure detailed elsewhere. The cleaned glass substrates were then placed into the deposition chamber and flushed with argon for 1 min to produce an oxygen-free surface. One milliliter of terpinen-4-ol monomer was used for every deposition. The films were fabricated at room temperature, pressure of 100 mTorr, and RF power of 25 W. In situ doping was performed by releasing the iodine vapours into the deposition chamber using the dopant inlet along with the monomer. Two different levels of doping were obtained by varying the amount of iodine placed into the flask, 50 mg and 150 mg of solid iodine for low- and high-level doping, respectively.

Thickness, roughness, and optical properties of the doped and pristine samples deposited on glass substrates were examined through variable angle spectroscopic ellipsometry (model M-2000D, J.A. Woollam Co. Inc., Lincoln, NE, USA) and UV-VIS spectroscopy (Avantes Avaspec-2048 spectrometer, Avalight-DHc light source, Avantes, Apeldoorn, The Netherlands). FTIR spectra of samples deposited onto KBr pallets were recorded at room temperature using a single beam FTIR spectrometer (Nicolet Maxim, Thermo Fisher Scientific, Waltham, MA, USA). X-ray photoelectron spectroscopy (XPS) survey spectra were collected using an Axis Ultra spectrometer (Kratos Analytical Ltd., Stretford, UK), equipped with a monochromatic X-ray source (Al *Kα*, *hν* = 1486.6 eV) operating at 150 W. An atomic force microscope (AFM) (NTEGRA Prima, NT-MDT, Moscow, Russia) operated in semi-contact mode was used to obtain images of the surface topography and to quantify the surface roughness. Surface wettability studies were performed using a contact angle system (KSV 101, CCD camera, KSV Instruments Ltd, Helsinki, Finland) employing the sessile drop method. An average of ten measurements per sample was obtained using sterilized nanopure H_2_O (18.2 M Ω·cm^−1^), diiodomethane (Sigma Aldrich, St. Louis, MO, USA) and ethylene glycol (Sigma Aldrich). The average contact angle for each liquid was used to calculate the surface free energy and its components, based on the Lewis acid/base method [47]. For *J*–*V* measurements, metal−insulator−metal (MIM) structures were prepared by depositing aluminium electrodes via thermal evaporation using a custom-made shadow mask placed beneath the samples.

## 4. Conclusions

Polyterpenol thin films were manufactured using RF plasma polymerisation. Iodine doping was carried out in situ, which led to significant changes in the chemical structure of the film. FTIR studies revealed that iodine was being incorporated into the backbone of the polymer, and that such incorporation resulted in the notable reduction in the methyl and methylene functional groups and appearance of C=C bond. Reduction in oxygen and increase in carbon atomic fractions as determined by XPS studies further indicated that abstraction of H, most probably by elimination of water, was one of the likely reaction pathways to take place during co-polymerisation. Higher levels of doping notably increased the refractive index of the thin films, from 1.54 to 1.70 at 500 nm wavelength, and significantly reduced the transparency of films, owing to the strong absorption of light characteristic of iodine species. Introduction of iodine impurity reduced the band gap of polyterpenol, by extending the density of states more into the visible region of the electromagnetic spectrum and an increase in C=C bonds in the iodine doped film layers compared to pristine polyterpenol. Doping increased the conductivity from 5.05 × 10^−8^ S/cm to 7.74 × 10^−7^ S/cm and 1.20 × 10^−6^ S/cm (at 20 V) for increasing doping levels. When subjected to annealing to 100 °C for 5 h, the band gap of doped polyterpenol increased slightly, the increase was attributed to desorption of loosely bonded iodine atoms from the surface of the film, and remained stable thereafter. Doping was found not to affect the surface morphology of the film. All films were smooth, defect-free, and uniform. Increased hydrophilicity of doped samples was attributed to a significantly larger number of polar functional groups exposed to the top of the polymer surface. Iodine doped samples were found to be monopolar in nature, largely manifesting electron-donor properties, although the electron-acceptor fraction of the surface tension for iodine doped samples was significantly higher than that of pristine polyterpenol, due to the presence of iodine atoms.

## Figures and Tables

**Table 1:** X-ray photoelectron spectroscopy (XPS) and Fourier transform infrared spectroscopy (FTIR) characterization of pristine and iodine doped polyterpenol samples.

Constituent	Pristine	Doped
*Atomic fractions* ^1^, %		
I	–	0.1
O	22.8	15.2
N	0.9	0.6
C	76.0	83.9
Si	0.3	0.2
*Chemical structure*	*Group frequency* ^2^, cm^−1^	
Hydroxy group, H-bonded OH stretch	~3460	3438
Methyl asymmetric C–H stretch	2955	2958
Methylene asymmetric C–H stretch	2930	2932
Methyl symmetric C–H stretch	2875	2874
Ketone	1708	1708
Alkenyl C=C stretch	–	1619
Methylene C–H bend	1457	1450
Methyl symmetric C–H bend	1377	1379
Skeletal C–C vibrations	~1020	~1180
Aliphatic C–I stretch	–	600–485

^1^ Based on XPS spectra; ^2^ Based on FTIR spectra.

**Table 2:** Band gap, direct allowed, and indirect allowed transition energy gap of pristine and iodine-doped polyterpenol thin films.

Sample Type	Band Gap, eV	Direct Allowed Transitions *E_g_*, eV	Indirect Allowed Transitions *E_g_*, eV
pristine polyterpenol	3.33	3.44	2.81
iodine-doped (I)	2.51	2.93	1.85
iodine-doped (II)	2.29	2.61	1.64
iodine-doped (II) annealed ^1^	2.48	2.82	1.74

^1^ Samples annealed to 100 °C for 5 h.

**Table 3:** Contact angle and surface tension for pristine and doped polyterpenol thin films fabricated at 25 W.

Surface Parameters	Pristine	Doped I	Doped II
*Surface roughness parameters* ^1^			
*R_max_*, nm	5.4 ± 1.6	6.1 ± 2.4	6.8 ± 2.7
*R_q_*, nm	0.38 ± 0.02	0.42 ± 0.05	0.44 ± 0.05
*R_a_*, nm	0.51 ± 0.06	0.56 ± 0.07	0.61 ± 0.08
*R_skw_*	0.22	0.51	0.76
*R_kur_*	1.65	2.13	2.51
*Contact angle*, °			
water	68.7 ± 1.6	27.8 ± 2.7	61.6 ± 2.0
glycerol	71.6 ± 1.8	44.4 ± 1.5	47.1 ± 1.7
DIM	38.1 ± 2.1	31.7 ± 1.9	28.4 ± 1.4
*Surface tension fractions*, mJ·m^−2^			
γ^LW^	40.5	23.5	23.9
γ^+^	0.3	2.35	5.6
γ^−^	19.8	57.3	14.2
γ^S^	45.1	46.7	41.2

^1^ Data for the 10 µm × 10 µm scanning area.
